# Multi-Camera Hierarchical Calibration and Three-Dimensional Reconstruction Method for Bulk Material Transportation System

**DOI:** 10.3390/s25072111

**Published:** 2025-03-27

**Authors:** Chengcheng Hou, Yongfei Kang, Tiezhu Qiao

**Affiliations:** 1 College of Physics and Optoelectronics Engineering, Taiyuan University of Technology, Taiyuan 030024, China; 2 China Energy Baotou Energy Co., Ltd., Baotou 014000, China; 11510472@ceic.com; 3 College of Integrated Circuits, Taiyuan University of Technology, Taiyuan 030024, China

**Keywords:** bulk material, multi-camera system, hierarchical calibration, three-dimensional reconstruction, speckle structured light

## Abstract

Three-dimensional information acquisition is crucial for the intelligent control and safe operation of bulk material transportation systems. However, existing visual measurement methods face challenges, including difficult stereo matching due to indistinct surface features, error accumulation in multi-camera calibration, and unreliable depth information fusion. This paper proposes a three-dimensional reconstruction method based on multi-camera hierarchical calibration. The method establishes a measurement framework centered on a core camera, enhances material surface features through speckle structured light projection, and implements a ‘monocular-binocular-multi-camera association’ calibration strategy with global optimization to reduce error accumulation. Additionally, a depth information fusion algorithm based on multi-epipolar geometric constraints improves reconstruction completeness through multi-view information integration. Experimental results demonstrate excellent precision with absolute errors within 1 mm for features as small as 15 mm and relative errors between 0.02% and 2.54%. Compared with existing methods, the proposed approach shows advantages in point cloud completeness, reconstruction accuracy, and environmental adaptability, providing reliable technical support for intelligent monitoring of bulk material transportation systems.

## 1. Introduction

Bulk material transportation systems are widely used in industrial production, and real-time acquisition of their three-dimensional (3D) geometric information is crucial for intelligent system control and safe operation. In recent years, with the rapid development of computer vision technology, vision-based 3D measurement methods have received widespread attention in the field of bulk material detection due to their advantages, such as non-contact and high efficiency [1,2].

Currently, 3D reconstruction of bulk material transportation systems mainly employs measurement technologies such as structured light [3,4], time of flight (TOF) [5,6], light detection and ranging (LiDAR) [7,8], and binocular vision [9,10,11]. Structured light technology enhances measurement accuracy through the active projection of coded patterns, but stripe distortion easily occurs on strongly scattering material surfaces [12]. TOF cameras have the advantage of good real-time performance but suffer from low spatial resolution and susceptibility to ambient light interference [13,14]. LiDAR uses laser beams for 3D imaging and is mainly used for medium-to-long range high-precision measurements [15]. In bulk material transportation systems, binocular vision has been widely applied due to its advantages of simple structure and low cost. Zhang et al. [16] proposed a binocular vision-based method for measuring large coal block volume, achieving volume calculation through 3D information obtained from binocular cameras. Wang et al. [17] proposed a coal weight detection system based on binocular vision and T-S fuzzy inference, utilizing local entropy transformation combined with K-means clustering segmentation and a watershed algorithm to achieve real-time monitoring of coal loading on conveyor belts. Xu et al. [18] constructed a sectional volume flow differential measurement model and dynamic bulk density estimation model by obtaining 3D point cloud data through line laser-assisted parallel binocular stereo matching combined with radio frequency identification (RFID) technology for conveyor belt section marking. However, existing binocular vision technology still faces severe challenges in practical applications, encompassing the lack of obvious texture features on bulk material surfaces leads to difficult feature matching, and limited system field of view can easily result in material occlusion, which seriously constrains the application effectiveness of binocular vision [19].

To overcome the limitations of binocular vision, multi-camera vision technology provides a new solution for 3D reconstruction of bulk material transportation systems due to its larger field of view, stronger occlusion resistance, and higher measurement accuracy [20,21]. Pai et al. [22] proposed a 3D reconstruction system based on multi-camera stereo vision, combining photogrammetric calibration and the adaptive pipeline seed growth algorithm to achieve high-precision 3D reconstruction in complex scenes. Lee et al. [23] proposed the multi-head attention refiner (MA-R) method that integrates the multi-head attention mechanism into U-Net structures, effectively improving multi-view 3D reconstruction performance. Deep learning methods [24,25,26,27,28] achieve end-to-end 3D shape inference and have made significant progress in various reconstruction forms, such as depth maps, voxels, point clouds, meshes, and implicit surfaces, providing new research directions for multi-view 3D reconstruction [29]. These studies provide important theoretical foundations for the industrial application of multi-camera systems. However, when applying multi-camera technology to bulk material transportation systems, existing methods still face the following key challenges: First, bulk material surfaces lack obvious texture features, leading to poor stereo matching results based on traditional feature points, which seriously affects reconstruction accuracy. Second, the calibration process of multi-camera vision systems is complex, and traditional methods usually complete multi-camera calibration through the transmission of binocular calibration results, which can easily cause error accumulation and lacks the full utilization of geometric constraints between multiple cameras. Finally, in the process of multi-view depth information fusion, due to the complex surface morphology of bulk materials, the reliability of depth information obtained from different viewpoints varies greatly, and existing fusion methods struggle to ensure the completeness and accuracy of reconstruction results.

To address these issues, this paper proposes a 3D reconstruction method based on multi-camera hierarchical calibration, focusing on three key aspects: improving feature extraction reliability, optimizing system calibration strategy, and enhancing depth information fusion. The main innovations include the following:

- A multi-camera measurement theoretical framework centered on the core camera was established. The camera with the optimal field of view is selected as the core camera, a unified measurement coordinate system is constructed, and speckle structured light technology is combined to enhance material surface features, thereby improving the reliability of feature matching.
- A globally optimized hierarchical calibration strategy was proposed. A ‘monocular-binocular-multi-camera association’ hierarchical calibration method is adopted, which fully utilizes geometric constraints between multiple cameras. Through global optimization algorithms, the system calibration accuracy is improved, effectively reducing error accumulation.
- An algorithm based on multi-view depth information fusion was developed. By establishing multi-epipolar geometric constraints, multi-view disparity calculation is achieved, and reconstruction accuracy and completeness are improved through depth information fusion.

These innovations form a complete technical solution, providing a new method for the high-precision 3D reconstruction of bulk material transportation systems. Subsequent sections of this paper are arranged as follows: Section 2 details the basic theory and key technologies of multi-camera measurement; Section 3 verifies the feasibility and effectiveness of the proposed method through experiments; and Section 4 summarizes the work of this entire paper.

## 2. Materials and Methods

To address the challenges of indistinct surface features and unstable measurement accuracy in the 3D reconstruction of bulk material transportation systems, this paper proposes a 3D reconstruction method based on a multi-camera system. This method encompasses three key technologies: First, multi-camera measurement principles and imaging models are established, enhancing the identifiability of material surface features through speckle structured light enhancement technology. Second, a multi-camera hierarchical calibration and global optimization method is designed, which achieves high-precision system parameter calibration through a ‘monocular-binocular-multi-camera association’ hierarchical strategy and global optimization. Finally, a method based on multi-view depth information acquisition and fusion is proposed, and the system reconstruction accuracy is improved through multi-view constraints and depth information fusion. This section will elaborate on the basic principles and implementation methods of these three key technologies.

### 2.1. Multi-Camera Measurement Principles and Imaging Model

**Figure 1:**
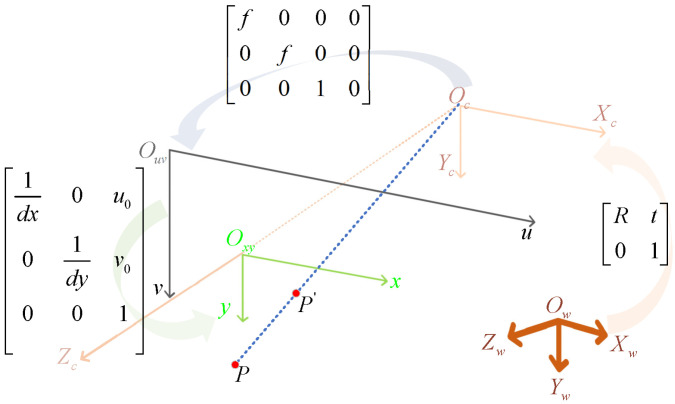
Coordinate system transformation relationship.

Multi-camera measurement systems achieve more efficient and accurate acquisition of target 3D information through the collaborative work of multiple cameras. To fully understand the working principles of this system, it is necessary to first introduce the imaging principles of monocular cameras. As shown in Figure 1, the imaging process of a single camera involves projection transformations from world coordinate system $O_{w}-X_{w}Y_{w}Z_{w}$ to camera coordinate system $O_{c}-X_{c}Y_{c}Z_{c}$, then to image plane coordinate system $O_{xy}-xy$, and finally to pixel coordinate system $O_{uv}-uv$.

The mathematical model of this transformation can be expressed as

(1) $$\begin{array}{c} s\left[\begin{array}{c} u\\ v\\ 1 \end{array}\right]=\left[\begin{array}{ccc} \frac{1}{dx} & 0 & u_{0}\\ 0 & \frac{1}{dy} & v_{0}\\ 0 & 0 & 1 \end{array}\right]\left[\begin{array}{cccc} \begin{array}{c} f\\ 0\\ 0 \end{array} & \begin{array}{c} 0\\ f\\ 0 \end{array} & \begin{array}{c} 0\\ 0\\ 1 \end{array} & \begin{array}{c} 0\\ 0\\ 0 \end{array} \end{array}\right]\left[\begin{array}{cc} R & t\\ 0 & 1 \end{array}\right]\left[\begin{array}{c} x_{w}\\ y_{w}\\ z_{w}\\ 1 \end{array}\right]\\ =\left[\begin{array}{cccc} \begin{array}{c} a_{x}\\ 0\\ 0 \end{array} & \begin{array}{c} 0\\ a_{y}\\ 0 \end{array} & \begin{array}{c} u_{0}\\ v_{0}\\ 1 \end{array} & \begin{array}{c} 0\\ 0\\ 0 \end{array} \end{array}\right]\left[\begin{array}{cc} R & t\\ 0 & 1 \end{array}\right]\left[\begin{array}{c} x_{w}\\ y_{w}\\ z_{w}\\ 1 \end{array}\right]=M_{1}M_{2}X_{w}=MX_{w}\;, \end{array}$$

where *s* is the scale factor, dx and dy are pixel sizes on *u* and *v* axes, respectively, $M_{1}$ is the camera intrinsic matrix, $M_{2}$ is the camera extrinsic matrix, and *M* is the projection matrix. ${\left(u,v,1\right)}^{T}$ represents homogeneous coordinates in the pixel coordinate system, and ${\left(x_{w},y_{w},z_{w},1\right)}^{T}$ represents homogeneous coordinates in the world coordinate system. Since monocular cameras lose depth information during the imaging process and cannot directly obtain 3D spatial data of targets, it is necessary to introduce multi-camera systems to achieve the precise measurement of target depth information.

**Figure 2:**
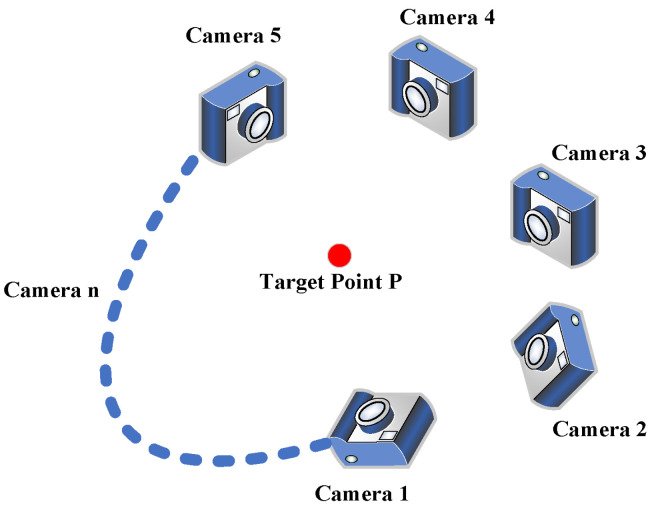
Multi camera vision system.

Multi-camera vision measurement is a 3D reconstruction method based on triangulation principles. Its core idea is to observe the same target from different viewpoints using two or more cameras and to reconstruct the target’s 3D spatial information by combining known camera parameters and image matching technology. For a multi-camera vision system composed of *n* cameras (as shown in Figure 2), assuming all cameras simultaneously capture a spatial target point *P*, for the *c*-th camera, the pixel coordinates $\left(u_{c},v_{c}\right)$ of point *P* satisfy Equation (1), as follows:

(2) $$s_{c}\left[\begin{array}{c} u_{c}\\ v_{c}\\ 1 \end{array}\right]=M_{c}X_{p}=\left[\begin{array}{cccc} \begin{array}{c} m_{11}^{c}\\ m_{21}^{c}\\ m_{31}^{c} \end{array} & \begin{array}{c} m_{12}^{c}\\ m_{22}^{c}\\ m_{32}^{c} \end{array} & \begin{array}{c} m_{13}^{c}\\ m_{23}^{c}\\ m_{33}^{c} \end{array} & \begin{array}{c} m_{14}^{c}\\ m_{24}^{c}\\ m_{34}^{c} \end{array} \end{array}\right]\left[\begin{array}{c} x_{p}\\ y_{p}\\ z_{p}\\ 1 \end{array}\right]\;,$$

where $m_{ij}^{c}$ is the element in the *i*-th row and *j*-th column of the projection matrix of camera *c*, $\left(x_{p},y_{p},z_{p}\right)$ represents the spatial coordinates of point *P*, and considering the influence of camera distortion, the pixel coordinates $\left(u_{c},v_{c}\right)$ are the values after distortion correction [30]. Expanding the above formula yields the following system of equations:

(3) $$\left\{\begin{array}{c} \left(u_{c}m_{31}^{c}-m_{11}^{c}\right)x_{p}+\left(u_{c}m_{32}^{c}-m_{12}^{c}\right)y_{p}+\left(u_{c}m_{33}^{c}-m_{13}^{c}\right)z_{p}=m_{14}^{c}-u_{c}m_{34}^{c}\\ \left(v_{c}m_{31}^{c}-m_{21}^{c}\right)x_{p}+\left(v_{c}m_{32}^{c}-m_{22}^{c}\right)y_{p}+\left(v_{c}m_{33}^{c}-m_{23}^{c}\right)z_{p}=m_{24}^{c}-v_{c}m_{34}^{c} \end{array}\right.\;.$$

Both of the above two equations are linear equations and contain three unknowns: $x_{p}$, $y_{p}$, and $z_{p}$. Therefore, the 3D coordinates of spatial target point *P* cannot be calculated through projection methods using a single camera. However, when using two or more cameras, the number of equations in the above equation group can be increased to four or 2n(n>2) equations, while the number of unknowns remains three. Considering the effects of environmental occlusion, data noise, and data loss, using *n* cameras can effectively reduce the random errors of monocular/binocular cameras. The 3D spatial coordinates $\left(x_{p},y_{p},z_{p}\right)$ of point *P* can be calculated by processing the 2n equations corresponding to *n* cameras through the least squares method. This is the basic principle of how multi-camera imaging achieves 3D reconstruction.

Based on the above principles, implementing 3D reconstruction measurement methods based on multi-camera vision involves the following key steps: First, each camera needs to be calibrated to obtain its intrinsic matrix (including focal length, principal point coordinates, etc.) and distortion parameters. Then, multi-view calibration determines the relative position relationships between cameras, i.e., the extrinsic matrices (including rotation matrices and translation vectors). During actual measurement, all cameras synchronously capture target images, and corresponding points between images are found through feature extraction and matching algorithms. Subsequently, overdetermined equation systems [31] are established using camera parameters and matched corresponding points, and target point 3D coordinates are solved through the least squares method. Finally, target 3D morphology reconstruction is completed by combining sufficient 3D points. This method significantly improves 3D reconstruction accuracy and robustness through the collaborative work of multiple cameras, effectively overcoming the limitations of monocular or binocular systems in complex environments.

However, the visual feature extraction of bulk materials faces numerous challenges in industrial field environments. First, bulk materials themselves often present dark or monotone colors with small color differences from conveyor belts, which makes it difficult for traditional passive multi-view vision systems to accurately identify material surface features. Second, unstable lighting conditions and ambient light interference in industrial environments further degrade image quality, leading to unreliable matching results.

To solve these problems, this paper introduces speckle structured light to enhance the imaging quality of multi-camera system. Structured light patterns are mainly divided into line and area-based methods. Although line-based structured light offers higher precision, it requires time-consuming line-by-line scanning. Similarly, grating fringe techniques need multiple sequential phase patterns, making them complex and vulnerable to object movement—unsuitable for online 3D reconstruction. In contrast, speckle structured light, as an area-based technique, requires only a single projected pattern for 3D reconstruction, offering advantages in measurement efficiency and interference resistance. Computer-simulated digital speckle patterns offer simplicity compared to laser speckles and artificial spray patterns, with the added benefit of flexible parameter adjustment based on target characteristics.

The proposed method utilizes a global randomly distributed speckle generation technique with special markers. This approach adopts a spatial gridding strategy with appropriate distance constraints to ensure both randomness and uniformity in speckle distribution, effectively avoiding the speckle clustering common in traditional local random distribution methods. Within an M×N image plane, the position coordinates $\left(x_{i},y_{i}\right)$ of each speckle point are generated through a uniform random distribution as follows:

(4) $$\left\{\begin{array}{c} x_{i}=M*rand\left[0,1\right]\\ y_{i}=N*rand\left[0,1\right] \end{array}\right.\;.$$

To prevent feature overlap from excessively dense speckles, the Euclidean distance between each newly generated speckle point $p_{i}\left(x_{i},y_{i}\right)$ and all existing points is calculated as follows:

(5) $$d\left(p_{i},p_{j}\right)=\sqrt{{\left(x_{i}-x_{j}\right)}^{2}+{\left(y_{i}-y_{j}\right)}^{2}}\;.$$

A new point is accepted only when $d(p_{i},p_{j})$ exceeds the minimum allowable distance $d_{\min}$ between speckle points. This distance constraint mechanism ensures appropriate spacing between speckle points, avoiding feature overlap while maintaining uniform feature density.

To improve the recognition and matching efficiency of the structured light pattern, the method implements a region pre-segmentation strategy with 16 special marker points. The M×N image plane is divided into 4 × 4 blocks, with each block’s center point serving as a special marker point $p_{s}\left(x_{s},y_{s}\right)$. The center coordinates of marker points are determined by

(6) $$\left\{\begin{array}{c} x_{s\_i}=M/4\times \left(i+0.5\right)\\ y_{s\_j}=N/4\times \left(j+0.5\right) \end{array}\right.\;,$$

where i,j=0,1,2,3. This block-center-based marker arrangement ensures spatial distribution uniformity, facilitating rapid localization and matching of corresponding regions during subsequent image processing. Considering camera installation angle effects, region pre-segmentation uses proportional division: first, it extracts marker feature points from the speckle structured light image, and then it divides regions using the mean value between adjacent feature points. Additionally, feature point distance interval thresholds are set; for missing feature points exceeding the threshold, region pre-segmentation can be completed using other points in the same row or column.

**Figure 3:**
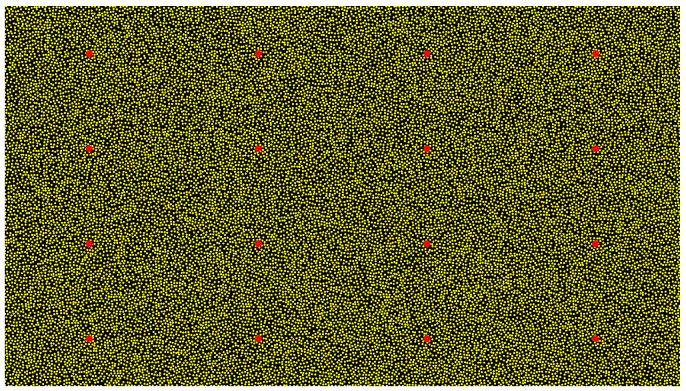
Speckle structured light.

As shown in Figure 3, the generated speckle structured light uses a dark background to highlight feature points. The speckle points appear in bright gold, with 25,000 randomly distributed points maintained by global distance constraints, ensuring uniformity. Special marker points are bright red and 10 times larger than speckle points, arranged according to block-center rules. Using this speckle pattern with adjustable grayscale value ranges not only effectively enhances material surface feature information but also maintains good robustness under varying lighting conditions. This design combines high-density random speckles with regular special markers, ensuring sufficient feature information for precise matching while enabling rapid region pre-segmentation, significantly improving system efficiency while maintaining measurement accuracy.

### 2.2. Multi-Camera Hierarchical Calibration and Global Optimization

Traditional multi-camera calibration methods decompose multiple cameras into multiple binocular systems and achieve extrinsic calibration through matrix transmission. However, this method suffers from error accumulation, optimization difficulties, and limited practicality. To address these issues, this paper proposes a multi-camera hierarchical calibration method based on global optimization. This method decomposes the calibration problem into four levels: monocular calibration, binocular calibration, multi-camera association, and global optimization. The optimal core camera is selected to establish a unified coordinate system, and high-precision calibration is achieved by combining a complete geometric constraint system. This hierarchical strategy not only overcomes error accumulation problems but also reduces optimization difficulty while ensuring system geometric consistency and computational efficiency. The following sections detail each level’s content.

#### 2.2.1. Monocular Calibration

Monocular calibration is the fundamental step of the entire system calibration. Each camera needs to be calibrated individually to obtain its intrinsic parameters and distortion coefficients. In this work, Zhang’s calibration method [32] is employed for monocular calibration. Since this method is well established and widely adopted in the field of computer vision [33,34,35,36], the specific implementation details will not be elaborated in this paper.

#### 2.2.2. Binocular Calibration

Binocular calibration builds upon monocular calibration and aims to determine the geometric relationship between two cameras, which is key to implementing stereo vision. Through establishing epipolar geometric constraints [37] and spatial transformation relationships, it provides the necessary geometric foundation for 3D reconstruction and depth estimation.

In a multi-camera vision system, the camera with the best field-of-view coverage and optimal imaging quality is selected as the core camera $C_{k}$. This ensures sufficient field-of-view overlap between the core camera $C_{k}$ and the other n−1 cameras. The relative pose relationships between the core camera and each non-core camera, as well as between non-core cameras themselves, are calculated, respectively. Based on epipolar geometric constraints, mathematical models are established to solve the rotation matrix *R* and translation vector *t* between each pair of cameras.

**Figure 4:**
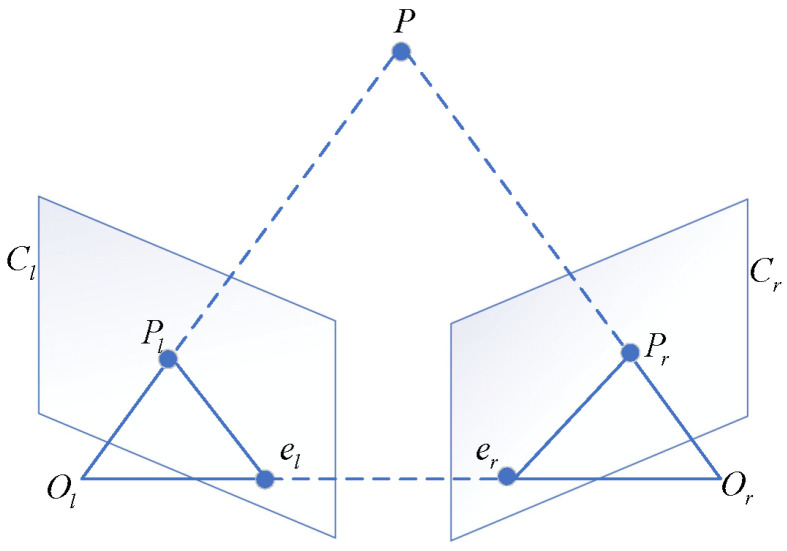
Binocular vision model.

As shown in Figure 4, for a spatial point *P* in the binocular camera system, its imaging points are $P_{l}$ and $P_{r}$, respectively ($P_{l}$ and $P_{r}$ are corresponding points), with camera optical centers $O_{l}$ and $O_{r}$. If the extrinsic parameters of the two cameras are known as $R_{l}$, $t_{l}$ and $R_{r}$, $t_{r}$, then the 3D spatial coordinates can be converted to coordinates $P_{l}$ and $P_{r}$ in their respective camera coordinate systems via the following formula:

(7) $$\left\{\begin{array}{c} P_{l}=R_{l}P+t_{l}\\ P_{r}=R_{r}P+t_{r} \end{array}\right.\;.$$

Using the coordinate system of camera $C_{l}$ as the reference coordinate system, the two equations in Equation (7) can be solved simultaneously to obtain the rotation matrix $R_{rl}$ and translation vector $t_{rl}$ of camera $C_{r}$ relative to camera $C_{l}$, as follows:

(8) $$\left\{\begin{array}{c} R_{rl}=R_{r}R_{l}^{-1}\\ t_{rl}=t_{r}-R_{r}R_{l}^{-1}t_{l} \end{array}\right.\;.$$

The pose relationship between binocular cameras can be obtained through Equation (8), thus completing stereo calibration. Multiple sets of images are used to ensure solution accuracy. Through this method, stereo calibration between the core camera and the other n−1 cameras is completed, resulting in a total of n−1 stereo calibrations. Simultaneously, stereo calibration between the other n−1 non-core cameras is also completed, resulting in an additional $C_{n-1}^{2}$ stereo calibrations.

#### 2.2.3. Multi-Camera Association

**Figure 5:**
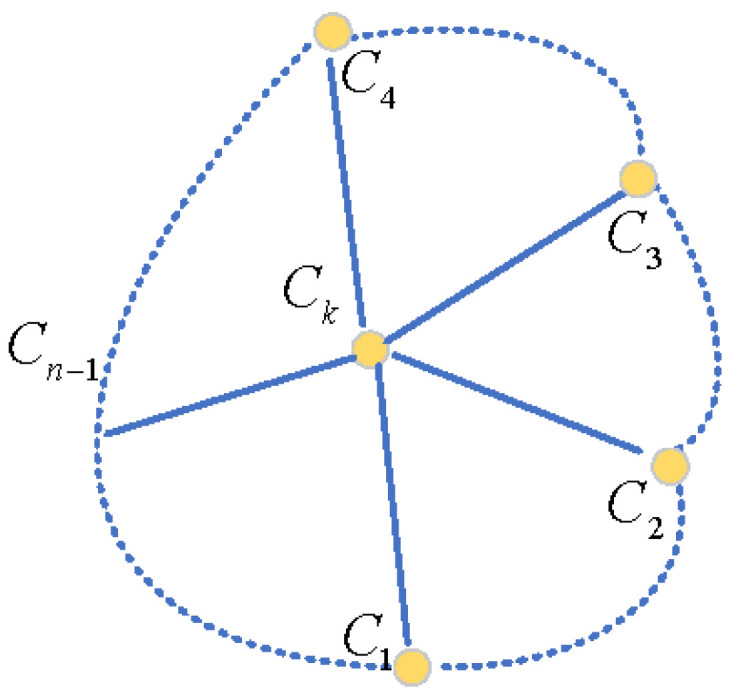
Multi-view vision system topology.

Multi-camera association is a fundamental task in constructing a multi-camera vision system. Its essence lies in solving the problem of transitioning from binocular calibration results to a unified multi-camera system, i.e., transforming from ‘fragmented to unified’. In this phase, the core camera serves as the reference. By integrating the relative poses obtained from the binocular calibration between the core camera and other cameras, as well as the relative poses between non-core cameras, a unified coordinate transformation network is established, forming a complete and geometrically consistent measurement system. This approach not only avoids the issue of error accumulation inherent in traditional methods but also fully utilizes the geometric redundancy within the system. Figure 5 illustrates the topological structure of the multi-camera vision system centered around the core camera $C_{k}$.

For any camera $C_{i}$, its transformation relative to core camera $C_{k}$ is

(9) $$T_{ki}=\left[R_{ki}\left|t_{ki}\right.\right],\;i=1,2,\cdots n-1\;,$$

where $T_{ki}$ is the direct transformation from core camera $C_{k}$ to camera $C_{i}$. The transformation relationship between any two non-core cameras $C_{i}$ and $C_{j}$ is

(10) $$T_{ij}=\left[R_{ij}\left|t_{ij}\right.\right],\;i\neq j\;.$$

Additionally, the transformation between $C_{i}$ and $C_{j}$ can also be obtained through transmission via core camera $C_{k}$, denoted as $T_{ij}^{*}$:

(11) $$T_{ij}^{*}=T_{ki}^{-1}\cdot T_{kj}=\left[R_{ki}^{-1}\left|-R_{ki}^{-1}t_{ki}\right.\right]\cdot \left[R_{kj}\left|t_{kj}\right.\right]\;.$$

According to the multiplication rules of homogeneous transformation matrix [38], the rotation matrix $R_{ij}^{*}$ and translation matrix $t_{ij}^{*}$ in $T_{ij}^{*}$ from Equation (11) are, respectively,

(12) $$\left\{\begin{array}{c} R_{ij}^{*}=R_{ki}^{-1}\cdot R_{kj}\\ t_{ij}^{*}=-R_{ki}^{-1}t_{ki}+R_{ki}^{-1}t_{kj}=R_{ki}^{-1}\cdot \left(t_{kj}-t_{ki}\right) \end{array}\right.\;.$$

Substituting Equation (12) into Equation (11) yields

(13) $$T_{ij}^{*}=\left[R_{ki}^{-1}\cdot R_{kj}\left|R_{ki}^{-1}\cdot \left(t_{kj}-t_{ki}\right)\right.\right]\;.$$

For cases where multiple transformation paths may exist between any two cameras, we typically choose the shortest path or the path through the core camera to minimize cumulative errors. However, in practical applications, due to measurement errors, transformation matrices obtained from Equations (10) and (13) may differ. This discrepancy arises from two sources: First, is a direct measurement error. The transformation relationship $T_{ij\_direct}$ obtained directly through binocular calibration contains measurement noise, which can be expressed as

(14) $$T_{ij\_direct}=T_{ij\_true}\cdot \exp\left(\xi^{\Lambda }\right)\;,$$

where $T_{ij\_true}$ is the true transformation relationship, and $\xi^{\Lambda }$ is the Lie algebra element [39] corresponding to measurement noise (the deviation is represented using Lie algebra for subsequent global optimization). The other source of difference is an indirect transmission error $T_{ij\_indirect}$. Although the shortest path (via the core camera) is chosen for the transformation, the transformation relationship still involves the propagation of two measurement errors. The indirect propagation error is given by

(15) $$T_{ij\_indirect}=T_{ki}^{-1}\cdot T_{kj}={\left[T_{ki\_true}\cdot \exp\left(\xi_{1}^{\Lambda }\right)\right]}^{-1}\cdot \left[T_{kj\_true}\cdot \exp\left(\xi_{2}^{\Lambda }\right)\right]\;,$$

where $\xi_{1}^{\Lambda }$ and $\xi_{2}^{\Lambda }$ are the noise terms from the two measurements, respectively.

To quantify the inconsistencies appearing in direct measurement and indirect transmission, the following error metrics are defined:

(16) $$\left\{\begin{array}{c} \varepsilon_{path}=∥T_{ij\_direct}-T_{ij\_indirect}∥\\ \varepsilon_{loop}=∥I-T_{ij}\cdot T_{ki}^{-1}\cdot T_{kj}∥ \end{array}\right.\;,$$

where $\varepsilon_{path}$ is the transformation path error, reflecting the difference between direct measurement values and indirect transformations obtained through the core camera; $\varepsilon_{loop}$ is the loop closure error, which ideally should satisfy $\varepsilon_{loop}=0$ for perfect loop closure. To address measurement errors in multi-view correlation calibration, we designed a global optimization-based calibration method for multi-camera vision systems.

#### 2.2.4. Global Optimization

Based on the above error analysis, the global optimization objective of the multi-camera system is to minimize the inconsistency between all direct measurements and indirect transformations. Define the set of optimization variables as the collection of transformation matrices of all cameras relative to the core camera as follows:

(17) $$X=\left\{T_{k1},T_{k2},\cdots ,T_{k(n-1)}\right\}\;.$$

The optimization objective function can be expressed as

(18) $$\min F\left(X\right)=\lambda_{1}E_{path}\left(X\right)+\lambda_{2}E_{loop}\left(X\right)=\lambda_{1}\sum_{ij}\varepsilon_{path}^{2}+\lambda_{2}\sum_{ij}\varepsilon_{loop}^{2}\;,$$

where $\lambda_{1}$ and $\lambda_{2}$ are weight coefficients balancing the two error terms.

For this nonlinear optimization problem, this paper adopts the Levenberg–Marquardt (L-M) algorithm [40]. The L-M algorithm achieves a smooth transition between the gradient descent method and Gauss–Newton method by introducing a damping factor, providing better convergence and stability [41,42]. The increment equation is solved iteratively as follows:

(19) $$\left(J^{T}J+\lambda I\right)\delta x=-J^{T}e\;,$$

where *J* is the Jacobian matrix [43] composed of derivatives of path errors and loop closure errors, *e* is the error vector, δx is the parameter increment, and λ is the damping factor (L-M parameter). The dynamic adjustment of λ is based on the gain ratio function:

(20) ρ=[F(X)−F(X+δx)]/[F(X)−L(X+δx)],

where $L\left(X\;+\;\delta x\right)$ is the linear approximation model in the optimization problem. When $\rho >\eta_{h}$, λ decreases; when $\rho <\eta_{l}$, λ increases (where $\eta_{h}$ and $\eta_{l}$ are the high and low confidence thresholds, respectively). This adaptive adjustment mechanism makes the algorithm similar to gradient descent when far from the optimal solution (larger λ), and it makes it similar to the Gauss–Newton method when close to the optimal solution (smaller λ).

After iterative computations with repeated adjustments, the optimization process stops when the error is reduced below a predefined precision threshold. At this point, the optimization result represents the optimal solution for the calibration model parameters.

### 2.3. Multi-View Depth Acquisition and Fusion

Multi-camera vision systems acquire scene information from different viewpoints through multiple cameras, providing richer geometric constraints and more reliable depth information compared to traditional binocular vision. Multi-camera stereo matching is the core step in achieving 3D reconstruction with multi-camera systems. The basic approach involves selecting a core camera as the reference and transforming the multi-camera matching problem into multiple stereo matching subproblems based on the core camera. In this work, a multi-camera system consisting of five cameras is constructed. This section focuses on the core camera-based multi-camera stereo matching method, including key techniques such as multi-epipolar rectification, disparity computation, and depth information fusion.

#### 2.3.1. Multi-Epipolar Rectification Based on Core Camera

**Figure 6:**
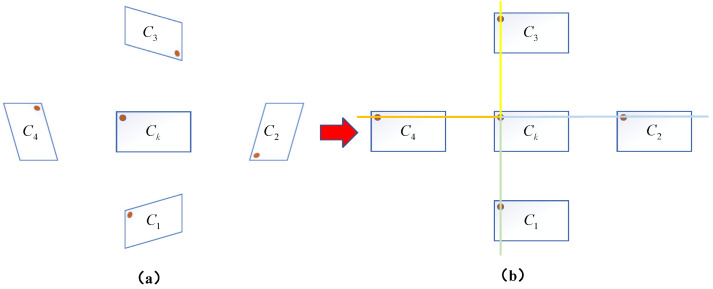
Results of multi-epipolar rectification based on the core camera: (**a**) Before rectification. (**b**) After rectification.

Multi-epipolar rectification based on the core camera is a crucial preprocessing step for achieving multi-camera stereo matching. In a five-camera vision system, a camera with a wide field of view and high imaging quality is selected as the core camera, while the other cameras serve as auxiliary cameras. The rectification of the multi-camera system is performed with the core camera as the reference, preserving the geometric properties of the core camera’s image with minimal distortion, while transforming the images of all auxiliary cameras to a position coplanar with the core camera. This rectification strategy simplifies the processing pipeline of the multi-camera vision system: The core camera’s image remains stable, providing a reference baseline; the consistency of multi-image rectification facilitates subsequent fusion. The transformation of auxiliary camera images to a coplanar position simplifies the stereo matching space. The steps for multi-epipolar rectification based on the core camera are as follows:

1. Obtain the calibration parameters of the core camera and each auxiliary camera, including the intrinsic matrix and the rotation matrices and translation vectors between the cameras, using the hierarchical calibration method described in Section 2.2.
2. Select the core camera and any auxiliary camera, referring to the binocular vision model shown in Figure 4. Use the Bouguet algorithm [44] to compute the new rotation matrix $R_{k}^{\prime }$ for the core camera and the new rotation matrix $R_{i}^{\prime }$ for the auxiliary camera. $R_{k}^{\prime }$ is chosen to minimize distortion in the core camera’s image, while $R_{i}^{\prime }$ ensures that the auxiliary camera is coplanar with the core camera.
3. Calculate projection matrices based on the new rotation matrices and original intrinsic parameters:
  (21) $$\left\{\begin{array}{c} M_{k}=M_{k1}R_{k}^{\prime }\\ M_{i}=M_{i1}R_{i}^{\prime } \end{array}\right.\;,$$
  where $M_{k1}$ and $M_{i1}$ are the original intrinsic parameters of the core camera and auxiliary camera, respectively.
4. Perform image transformation to project the original images onto the new image planes.
5. Repeat the above steps to complete the epipolar rectification between the core camera and all other cameras. The results of multi-epipolar rectification are shown in Figure 6. In Figure 6b, different colored lines illustrate the binocular epipolar rectification relationships between the core camera and each auxiliary camera.

Through the above rectification, the image pairs between the core camera and each auxiliary camera will satisfy the following conditions: corresponding points are strictly located on the same horizontal line, and the epipolar lines are parallel and horizontal. This core camera-based rectification strategy not only ensures the stability of the core view but also simplifies the subsequent stereo matching into a one-dimensional search problem, thereby improving matching efficiency and accuracy.

#### 2.3.2. Multi-View Disparity Calculation and Fusion

After completing epipolar rectification, disparity calculation is performed based on the core camera. In the binocular system formed by the core camera and each auxiliary camera, the positional difference between corresponding image points is constrained to the baseline direction, and this positional difference is referred to as disparity [45]. Through a coordinate transformation between subsystems, 3D reconstruction of the same physical point in different subsystems can be achieved, enabling the transfer of 3D point coordinates obtained from any subsystem to other subsystems. This allows for the acquisition of 3D points in the multi-camera stereo system through point fusion and filtering.

**Figure 7:**
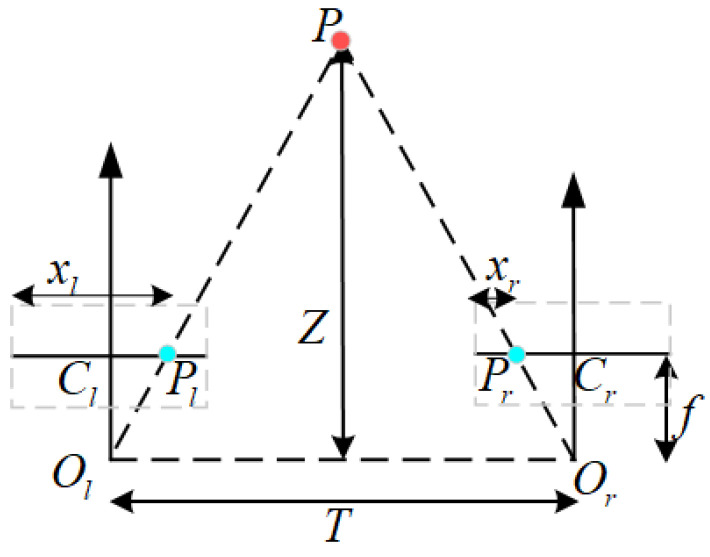
Relationship between binocular disparity and depth.

As shown in Figure 7, for a spatial point *P* and its projections $P_{l}$ and $P_{r}$ in the binocular camera system, the relationship between depth *Z* and disparity can be established based on the principle of similar triangles. For the rectified image pair, $P_{l}$ and $P_{r}$ are the imaging points (corresponding points) of point *P* on the left and right image planes, respectively. The perpendicular distance from point *P* to the binocular baseline is denoted as *Z*. The principal points of the left and right cameras are $C_{l}$ and $C_{r}$, respectively, and $x_{l}$ and $x_{r}$ are the horizontal coordinates of image points $P_{l}$ and $P_{r}$ on the left and right camera planes. *f* is the focal length of the cameras, which is the same for both cameras after rectification, and *T* is the baseline length. According to the principle of similar triangles, we have

(22) $$\frac{Z}{T}=\frac{Z-f}{T-\left(x_{l}-C_{l}\right)-\left(C_{r}-x_{r}\right)}=\frac{Z-f}{T-(x_{l}-x_{r})}\;,$$

where $C_{l}=C_{r}$, and the binocular disparity value is denoted as $d=x_{l}-x_{r}$. The depth information *Z* of spatial point *P* can then be calculated as

(23) $$Z=f\frac{T}{d}\;.$$

After obtaining the depth *Z*, the complete 3D coordinates of spatial point *P* can be calculated based on the pinhole camera model [46].

In the multi-camera system, the core camera and each auxiliary camera form independent disparity calculation units. To obtain reliable depth information, the system performs an arithmetic average of the depth values obtained from multiple viewpoints:

(24) $$Z_{final}=\frac{1}{n-1}\sum Z_{i}\;,$$

where *n* is the total number of cameras, and $Z_{i}$ is the depth estimate from each viewpoint.

Based on the obtained depth information, 3D reconstruction is performed using the core camera as reference. For each feature point in the observation scene of the bulk material transportation system, its 3D coordinates P(X,Y,Z) can be calculated by the following formula:

(25) $$\left\{\begin{array}{c} X=\left(x-C_{x}\right)*Z/f\\ Y=\left(y-C_{y}\right)*Z/f\\ Z=Z_{final} \end{array}\right.\;,$$

where (x,y) are the pixel coordinates of the feature point on the core camera’s image plane, $\left(C_{x},C_{y}\right)$ are the principal point coordinates of the core camera, and *f* is the focal length of the core camera. These parameters are obtained through camera calibration, and $Z_{final}$ is the average depth value calculated using Equation (24). The system processes each feature point within the field of view to obtain a complete depth map, achieving 3D reconstruction of the observation scene.

## 3. Experiments and Results Analysis

To validate the performance of the proposed multi-camera vision-based 3D reconstruction method for the transportation system, a multi-camera acquisition system was set up on a laboratory transportation system. The system captures image information of the conveyor belt and the bulk materials on it, and the proposed method is used to achieve 3D reconstruction of the bulk material transportation system. Finally, comparative experiments were conducted to compare the point cloud reconstruction results of the proposed method with those of the binocular 3D reconstruction, unstructured light multi-camera 3D reconstruction, and TOF camera 3D reconstruction.

### 3.1. Setup of the Multi-Camera System

**Figure 8:**
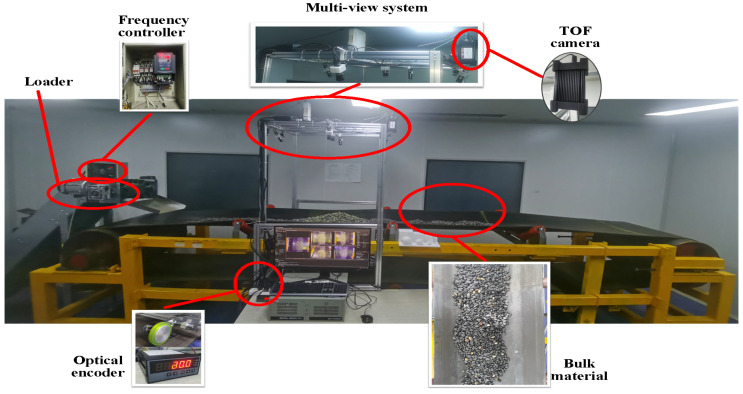
Conveyor system.

As shown in Figure 8, the laboratory conveyor system consists of a conveyor belt with a width of 0.8 m and a total length of 12 m. The conveyor uses three-trough idlers, with a distance of 0.78 m between the vertex points of the side idlers. The conveyor belt running speed can be adjusted through a frequency converter, with a maximum safe operating speed of 2 m/s. The system is equipped with traditional contact speed sensors (optical encoder) to measure the conveyor belt speed in real time. The proposed multi-camera system is mounted on the conveyor using a frame structure. Additionally, a TOF camera is installed on the system for comparative experiments.

**Figure 9:**
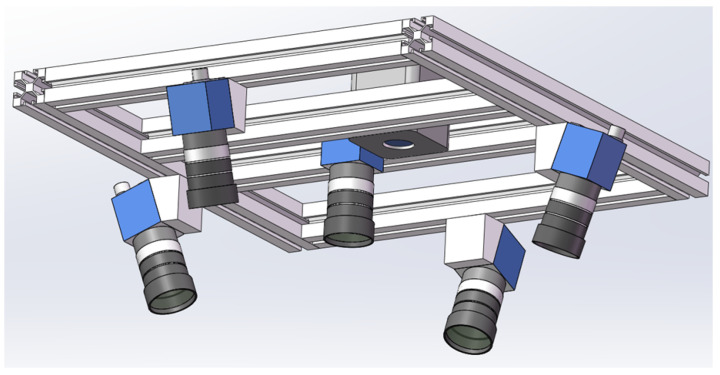
Multi-camera system.

In this study, considering the spatial constraints of the laboratory transportation system and the calibration complexity of the multi-camera, five cameras of the same model and configuration are used for image acquisition and 3D reconstruction, with a projector serving as the speckle structured light source. The deployment design of the cameras and projector is shown in Figure 9. To enhance the surface features of the measured bulk materials, speckle structured light is projected onto the surface of the target object using the projector, and images are captured simultaneously by the five cameras. The images captured by the five cameras show complete overlap of the conveyor belt and bulk materials. As seen in Figure 9, the core camera is positioned directly above the center of the conveyor system, while the other four non-core cameras are evenly distributed around the core camera at 90° intervals. The optical axes of the four non-core cameras are tilted toward the optical axis of the core camera. The projector, used to emit speckle structured light, is located beside the core camera. The tilt angles of the non-core cameras are adjustable, ensuring that the overlapping fields of view of the four cameras and the core camera fully cover the upper conveyor belt of the system, enabling complete 360-degree information acquisition of the bulk materials on the horizontal plane.

The model of the cameras used in this experiment is MER2-502-79U3C (DAHENG IMAGING, Beijing, China), equipped with a globally shuttered IMX250 CMOS sensor (SONY, Tokyo, Japan), and they transmit image data via a USB 3.0 interface. The lenses used in the experiment are HN-0828-6M-C2/3B (DAHENG IMAGING, Beijing, China) fixed-focus lenses. Additionally, to achieve synchronized acquisition across multiple cameras, all cameras are set to hardware trigger mode, and a synchronization trigger board is designed for the multi-camera system. The main parameters of the cameras and lenses are listed in Table 1.

To ensure complete coverage of the conveyor belt and bulk materials in the transportation system, the linear distance between the vertices of the bilateral idlers of the conveyor system is set as *w*, and the distance from the core camera lens to the vertices of the bilateral idlers is *h*. The field of view (FOV) of the core camera is α×β (i.e., $57^{\circ }\times 44.2^{\circ }$). The installation height *h* satisfies the following formula:

(26) $$h\geq \frac{w}{2}\cdot \frac{\alpha }{2}=\frac{0.78}{2}\cdot \frac{57}{2}=0.7183\;.$$

**Figure 10:**
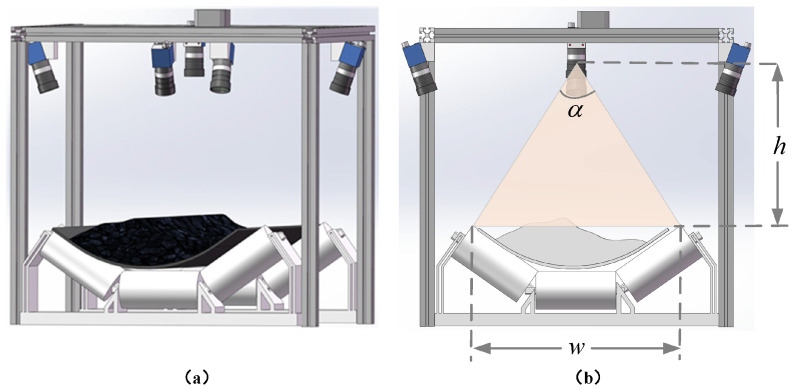
Installation diagram of the multi-camera system: (**a**) Overall installation view. (**b**) Front sectional view.

At this height, the core camera’s acquisition field of view on the plane of the bilateral idler vertices can reach 0.78 m × 0.58 m (where 0.78 m is the conveyor belt width direction and 0.58 m is the conveyor belt running direction), ensuring complete coverage of the conveyor belt area. Figure 10 shows the installation diagram of the multi-camera system.

**Figure 11:**
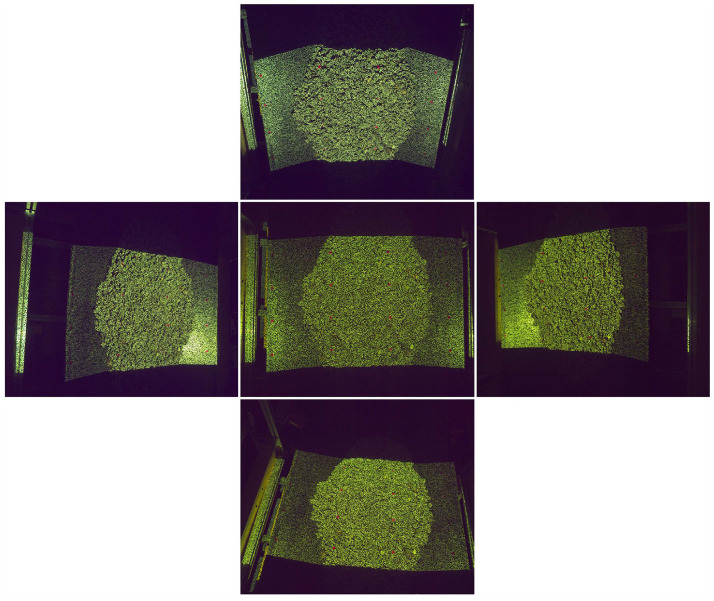
Images captured by multi-camera system.

Images captured by the multi-camera system of the transportation system are shown in Figure 11, with the central image being captured by the core camera.

### 3.2. Multi-Camera 3D Reconstruction Accuracy Measurement Experiment

**Figure 12:**
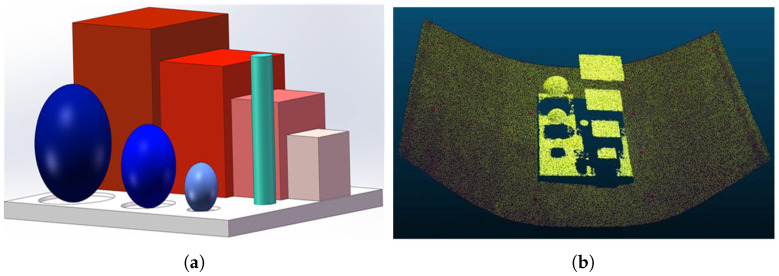
Multi-camera 3D reconstruction accuracy measurement: (**a**) Calibration module. (**b**) Three-dimensional reconstruction point cloud of the calibration module.

To test the accuracy of the proposed multi-camera vision 3D reconstruction method, a calibration module with multiple geometric features was designed. As shown in Figure 12a, the calibration module mainly consists of a base, four cubes of different sizes, three spheres of different diameters, and a cylinder. The base measures 285∗200∗10 mm, the four cubes have edge lengths of 100 mm, 80 mm, 60 mm, and 40 mm, respectively, the three spheres have diameters of 70 mm, 50 mm, and 30 mm, respectively, and the cylinder has a diameter of 20 mm and a height of 90 mm. The calibration module was fabricated using 3D printing, with a module precision of ±0.2 mm. The calibration module was placed on the conveyor belt, and the proposed multi-camera vision 3D reconstruction method was used to generate the point cloud of the module, as shown in Figure 12b. The point cloud software CloudCompare (V 2.13.2) was used to segment and fit the geometric features in the point cloud, measuring the dimensions of each geometric feature. The experimental results of the accuracy test are shown in Table 2.

From Table 2, it can be seen that the proposed multi-camera vision 3D reconstruction method demonstrates excellent measurement precision with a clear size-dependent characteristic: as target size increases, relative error decreases. For medium to large features (≥60 mm), such as the 100 mm and 80 mm cube edge lengths, the relative errors are merely 0.61% and 0.79%, respectively. On the other hand, for medium-sized features (20–60 mm), the relative errors range from 1.69% to 2.54%. Even for the smallest 30 mm sphere (15 mm radius), the absolute error is only 0.38 mm. The measurement accuracy of spheres slightly decreases as the diameter reduces, suggesting that curvature affects reconstruction precision. The base plate, being the largest planar structure, shows length and width measurement errors of 1.22% and 0.99%, respectively, while the height difference measurement between adjacent cubes shows a 2.10% error.

The cylinder height measurement has the largest relative error of 4.14%, which may be attributed to the high curvature of the small-diameter cylinder surface. Computational errors may cause its shape to be exaggerated into an irregular surface during 3D reconstruction, thereby affecting height measurement. In contrast, the diameter measurement of the same cylinder has an error of only 1.53%. Overall, the system maintains absolute measurement errors within 1 mm even for minimum feature sizes of 15 mm, with a maximum relative error of 2.54% (excluding the cylinder height). For bulk materials commonly found in transportation systems, this system fully meets industrial application accuracy requirements and provides reliable technical support for high-precision volume measurement.

### 3.3. Comparison Experiment Between Multi-Camera Vision and Binocular Vision

**Figure 13:**
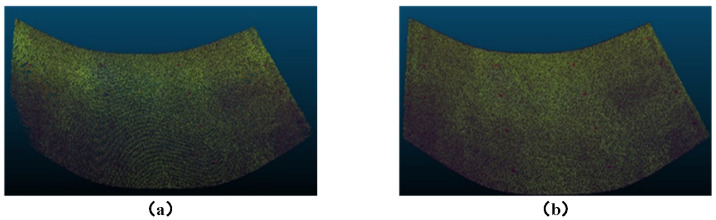
Three-dimensional reconstruction results for empty conveyor belt: (**a**) Binocular vision. (**b**) Proposed method.

**Figure 14:**
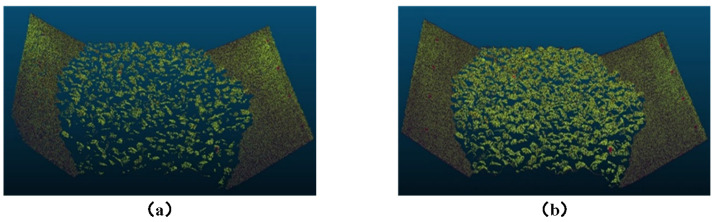
Three-dimensional reconstruction results for conveyor belt with bulk materials: (**a**) Binocular vision. (**b**) Proposed method.

**Figure 15:**
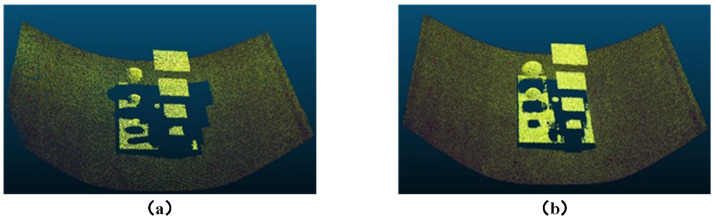
Three-dimensional reconstruction results for conveyor belt with calibration module: (**a**) Binocular vision. (**b**) Proposed method.

Three-dimensional reconstruction experiments were conducted on the conveyor belt using both the horizontal binocular setup from the multi-camera vision system shown in Figure 9 and the proposed multi-camera vision 3D reconstruction method. The reconstruction was performed for three scenarios: empty conveyor belt, loaded conveyor belt, and the conveyor belt with the calibration module placed on it. The resulting point cloud comparison diagrams are shown in Figure 13, Figure 14 and Figure 15. From the results, it can be observed that the traditional binocular method has poor reconstruction accuracy, with many error points and holes, and it fails to reconstruct the edge regions of the conveyor belt. Particularly in the case of reconstructing the loaded conveyor belt, the holes in the bulk materials on the conveyor belt are more severe, making it difficult to fully describe the continuity of the piled materials. In contrast, the proposed core camera-based multi-camera vision method significantly improves the reconstruction accuracy compared to the binocular method, with only occasional error points at object edges, but the number of error points is much smaller than that of the binocular method. Additionally, when the calibration module is placed on the conveyor belt, the binocular method suffers from significant occlusion, resulting in poor reconstruction of the module’s base plate.

The above comparative experiments demonstrate that the multi-camera approach offers significant advantages over binocular vision for bulk material reconstruction. Although this study focused on the comparison between multi-camera and binocular systems, the optimal number of cameras depends on specific application requirements, accuracy goals, and environmental constraints. In bulk material transportation systems, such as conveyor belts, several key factors influence the choice of a camera’s configuration: (1) spatial constraints, as the limited installation space above the conveyor restricts the number of cameras that can be feasibly deployed; (2) cost-effectiveness, as increasing the number of cameras introduces higher hardware and computational costs; and (3) system complexity, particularly in terms of calibration and synchronization, which become more challenging with additional cameras. For simpler scenarios, such as flat material surfaces, a three-camera system may suffice, while more complex scenarios may benefit from additional cameras if space and budget permit.

### 3.4. Comparison Experiment Between Multi-Camera Speckle Structured Light and Multi-Camera Non-Structured Light

**Figure 16:**
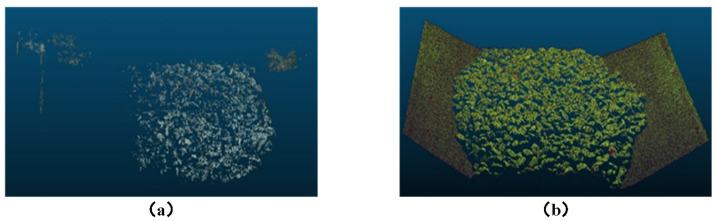
Comparison of 3D reconstruction results with and without structured light: (**a**) Non-structured light. (**b**) Structured light.

To verify the impact of speckle structured light on 3D reconstruction, this section compares the 3D reconstruction results of the conveyor belt and its bulk materials using multi-camera vision systems with and without speckle structured light. The experiments were conducted under the same environmental conditions and camera parameter settings. The experimental results are shown in Figure 16.

Since the surface of the conveyor belt is a textureless black belt, the images captured directly contain few feature points in the conveyor belt region. For the multi-camera vision system without structured light, 3D reconstruction of the empty conveyor belt was attempted, but repeated adjustments failed to generate a corresponding 3D point cloud; thus, it is not shown in Figure 16. In the 3D reconstruction results of the loaded conveyor belt, only a very small portion of the conveyor belt region was reconstructed. Comparing the structured light and non-structured light approaches, the structured light approach significantly improves both point cloud density and reconstruction accuracy, with the point cloud density in the material region increasing by approximately 9.5 times. The improvement is most noticeable for the empty conveyor belt, which has fewer surface features. In complex object regions, such as the edges and occluded areas of bulk materials, the structured light approach demonstrates better reconstruction stability.

### 3.5. Comparison Experiment Between Multi-Camera 3D Reconstruction and TOF Camera 3D Reconstruction

**Figure 17:**
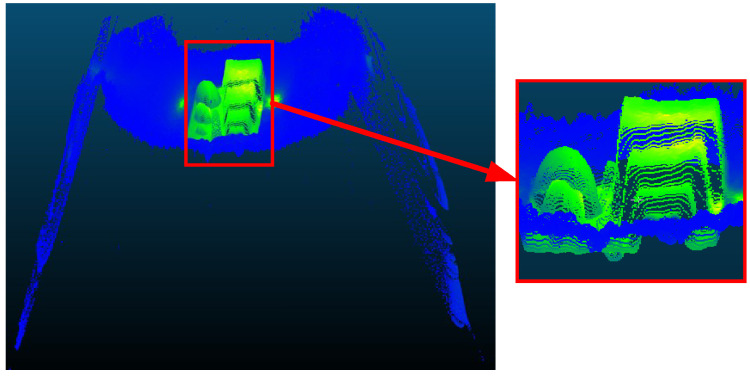
Point cloud acquired by TOF camera.

To comprehensively evaluate the performance of the proposed multi-camera 3D reconstruction method, comparative experiments were conducted with an industrial TOF camera. The TOF camera used in the experiment is the OI-G1480-R (Odos Imaging, Edinburgh, Scotland), with a resolution of 640 × 480, a field of view of $43^{\circ }\times 33^{\circ }$, and a maximum measurement distance of 6 m. The TOF camera was used to capture the calibration module, and the directly acquired point cloud image is shown in Figure 17.

From Figure 17, it can be observed that the standard geometric shapes captured by the TOF camera are deformed, especially the spheres, which have become ellipsoidal, and the cylinder, which has taken on a conical shape. Using CloudCompare, the geometric features in the point cloud were segmented and fitted, and the dimensions of each geometric feature were measured. The results are shown in Table 3. From the table, it can be seen that the relative errors of the 3D reconstruction using the TOF camera are relatively large. Particularly for the reconstruction of spherical/circular objects, the shapes are significantly distorted. For the cylinder diameter measurement, since the reconstruction result has become conical, the diameter of the base circle is larger, making it impossible to accurately reflect the actual dimensions. Therefore, the measured values are not annotated.

It is worth noting that the cylinder height measured by the TOF camera in Table 3 (91.978 mm) appears closer to the reference value (90 mm) than the multi-camera method result (86.2748 mm). However, this comparison is misleading. The enlarged view in Figure 17 clearly shows that the TOF camera’s point cloud extends beyond both the calibration module base plate boundaries and the conveyor range, contradicting physical reality. During geometric feature segmentation in CloudCompare, the measured cylinder height erroneously includes points that extend below the base plate. In contrast, the multi-camera method accurately preserves the geometric relationship between the cylinder and base plate, producing measurements that better represent the actual physical structure.

Comparing the performance of the proposed multi-camera vision 3D reconstruction method with that of the TOF camera, although the TOF camera has the advantage of fast data acquisition in certain scenarios, the proposed multi-camera vision 3D reconstruction method demonstrates significant advantages in terms of accuracy, environmental adaptability, and cost-effectiveness, making it more suitable for industrial applications with high precision requirements, such as conveyor belt systems.

## 4. Conclusions

This paper addresses the critical technical requirement of 3D reconstruction for bulk material transportation systems and systematically investigates a measurement method based on multi-camera vision. The proposed multi-camera vision measurement framework incorporates speckle structured light enhancement technology for surface feature extraction, and it employs a hierarchical calibration strategy of ‘monocular-binocular-multi-camera association’ with global optimization to improve system calibration accuracy.

Experimental results validate the feasibility and effectiveness of the proposed method: measurements of standard geometric objects demonstrate excellent precision with absolute errors within 1 mm for features as small as 15 mm and relative errors between 0.02% and 2.54% for most geometric features, fully meeting the accuracy requirements for bulk material measurement applications. Compared to traditional binocular vision, the proposed method significantly improves point cloud completeness and effectively addresses reconstruction issues in occluded and edge regions. Compared to non-structured light solutions, the point cloud density is increased by approximately 9.5 times. Compared to TOF cameras, the proposed method has clear advantages in geometric shape preservation and measurement accuracy. These results indicate that the proposed method can meet the engineering requirements for the 3D reconstruction of bulk material transportation systems.

Future work will primarily focus on optimizing real-time system performance and developing deep learning-based feature processing methods to enhance reconstruction accuracy and efficiency. Additionally, we will explore adaptive strategies to improve system robustness and adaptability to different spatial constraints and material characteristics in complex industrial environments. To further validate the advantages of the proposed method, comparative experiments with commercial 3D reconstruction systems, such as LiDAR and structured light, will also be conducted. These efforts aim to advance the practical application of this technology in bulk material transportation systems.

## Figures and Tables

**Table 1:** Main parameters of experimental camera and lens.

MER2-502-79U3C		HN-0828-6M-C2/3B	
Resolution	2448 (H) × 2048 (V)	Sensor size	2/3″
Sensor format	2/3″	Focal length (mm)	8
Pixel size	3.45 μm × 3.45 μm	F/No	F2.8–F16
Frame rate	79.1 fps	Angle of view (D × H × V)	68.5° × 57° × 44.2°
Exposure time	UltraShort: 1 μs–100 μs.		
	Standard: 20 μs–1 s.		
Synchronization	Hardware trigger, software trigger		

**Table 2:** Multi-camera 3D reconstruction accuracy measurement results.

Measurement Parameter	Actual Value (mm)	Measured Value (mm)	Relative Error (%)
100 mm cube edge length	100	100.6125	0.61
80 mm cube edge length	80	80.6290	0.79
60 mm cube edge length	60	60.0137	0.02
40 mm cube edge length	40	39.1015	2.25
70 mm sphere radius	35	34.1970	2.29
50 mm sphere radius	25	24.5766	1.69
30 mm sphere radius	15	14.6196	2.54
Cylinder diameter	20	20.3059	1.53
Cylinder height	90	86.2748	4.14
Base length	285	281.528	1.22
Base width	200	201.987	0.99
Height difference between adjacent cubes	20	19.5795	2.10

**Table 3:** TOF Camera 3D reconstruction results.

Measurement Parameter	Actual Value (mm)	Measured Value (mm)	Relative Error (%)
100 mm cube edge length	100	108.1152	8.12
80 mm cube edge length	80	82.5438	3.18
60 mm cube edge length	60	61.6870	2.81
40 mm cube edge length	40	44.4484	11.12
70 mm sphere radius	35	28.1255	19.64
50 mm sphere radius	25	19.2265	23.09
30 mm sphere radius	15	12.0833	19.44
Cylinder diameter	20	-	-
Cylinder height	90	91.978	2.20
Base length	285	293.286	2.91
Base width	200	205.293	2.65
Height difference between adjacent cubes	20	18.6571	6.71

## Data Availability

The data presented in this study are available upon request from the first and corresponding author.
